# Supplementary figures and images for: Uncovering the role of traditional Chinese medicine in immune-metabolic balance of gastritis from the perspective of Cold and Hot: Jin Hong Tablets as a case study
Source: Chin Med. 2024 Oct 4;19:134. doi: 10.1186/s13020-024-00998-8 (PMC11451182; doi:10.1186/s13020-024-00998-8)

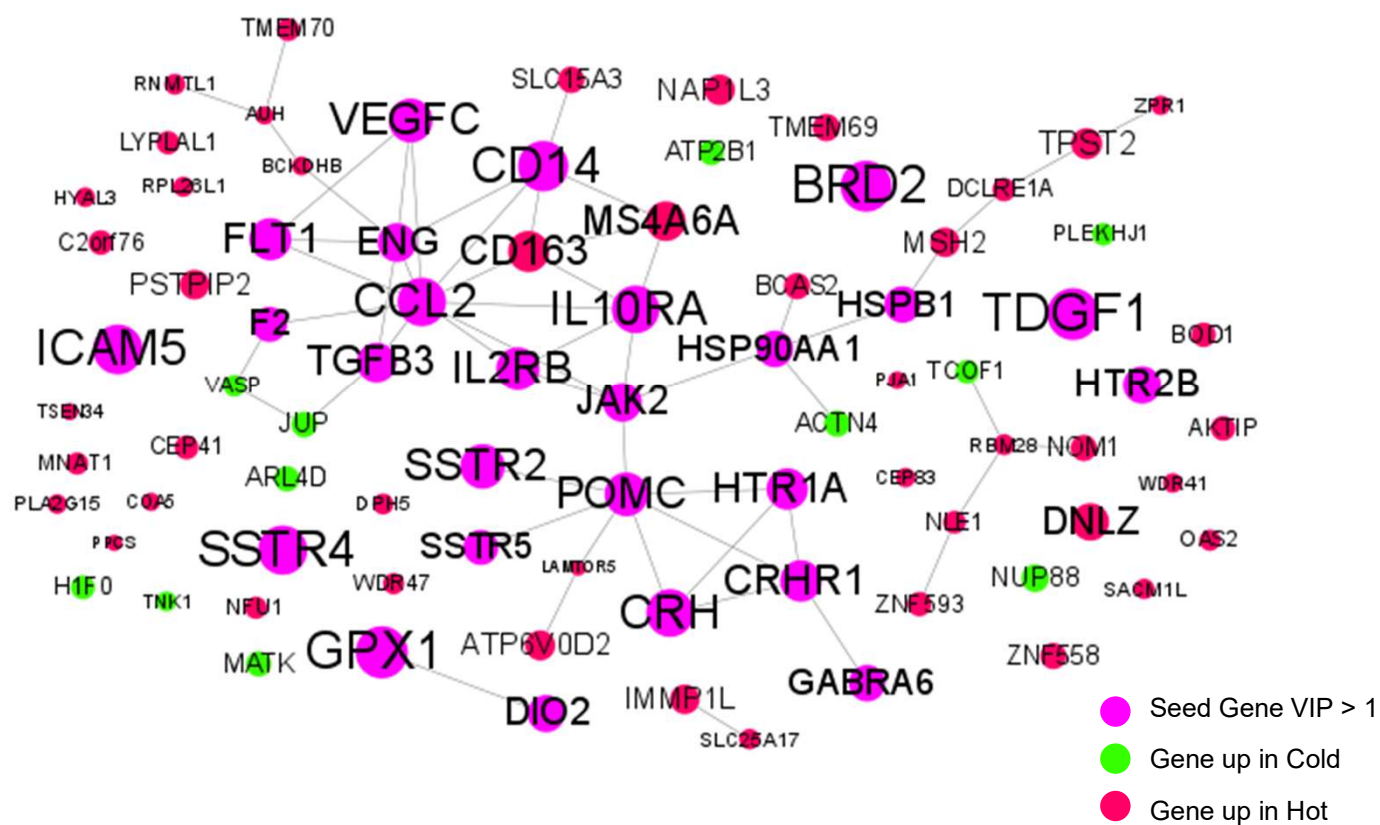

Figure S1

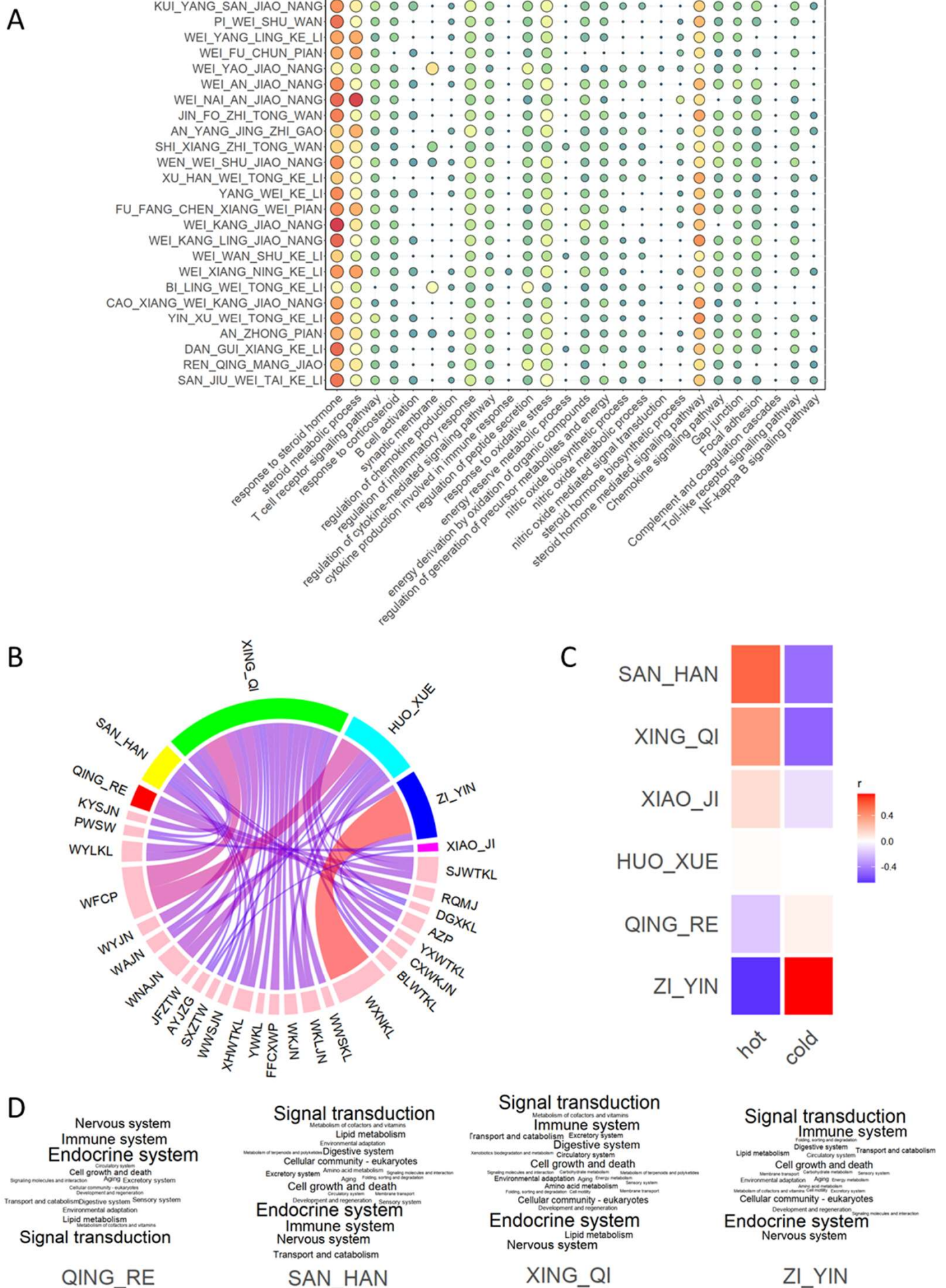

Figure S2

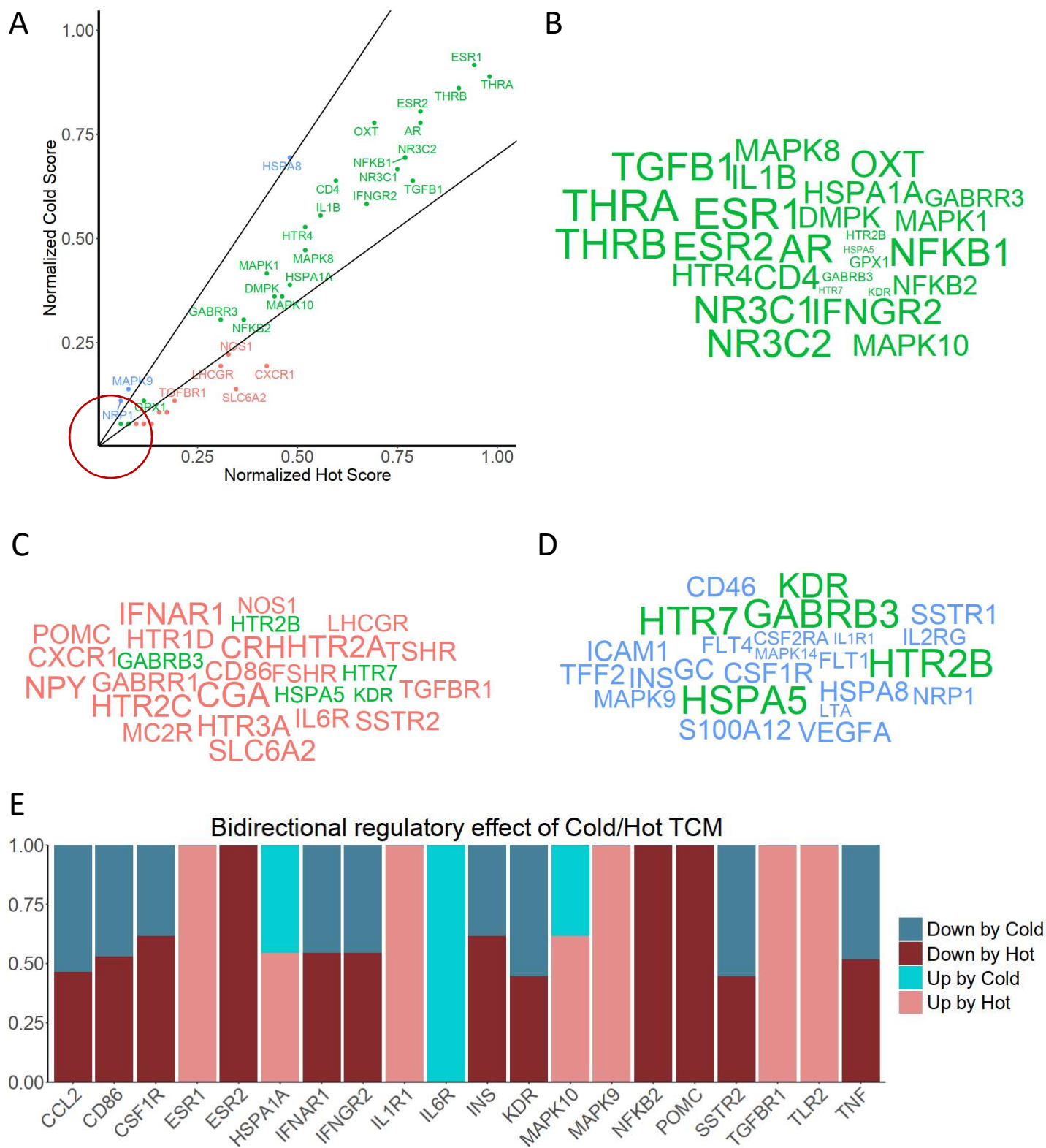

Figure S3

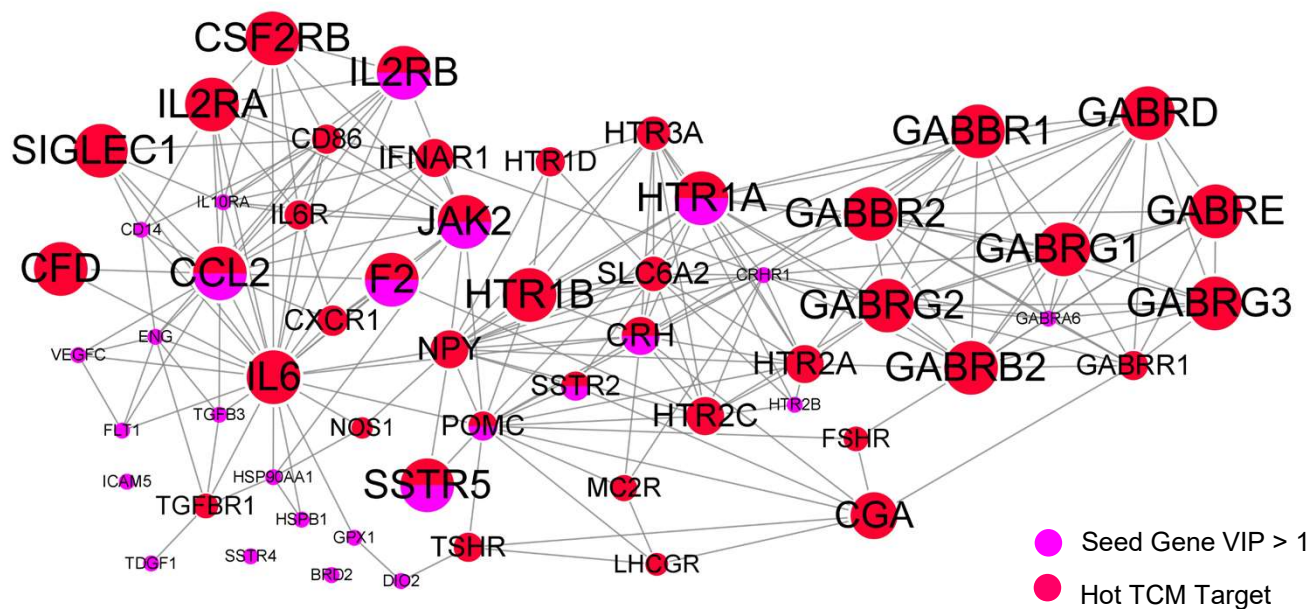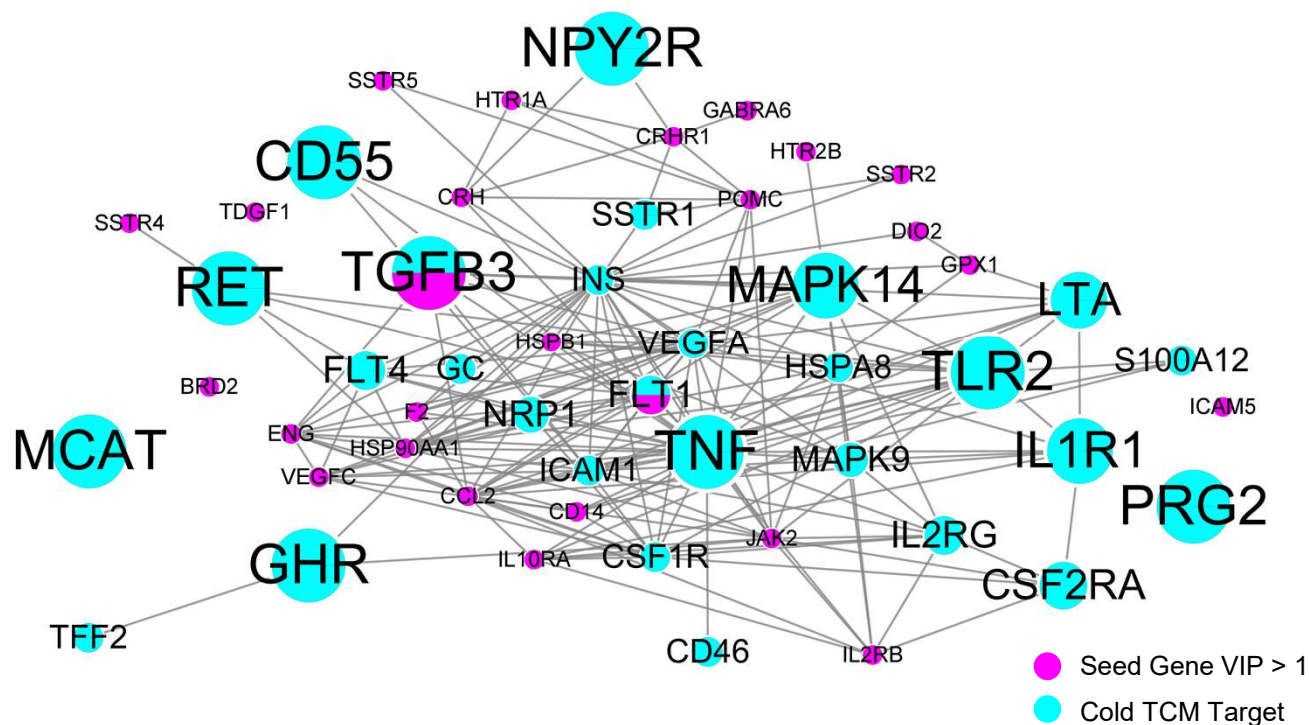

Figure S4

Supplement: Supplementary file 1 — Supplementary Material 1. [file 13020_2024_998_MOESM1_ESM.pdf]
